# On Propagation of Excitation Waves in Moving Media: The FitzHugh-Nagumo Model

**DOI:** 10.1371/journal.pone.0004454

**Published:** 2009-02-12

**Authors:** Elena A. Ermakova, Emmanuil E. Shnol, Mikhail A. Panteleev, Andrey A. Butylin, Vitaly Volpert, Fazoil I. Ataullakhanov

**Affiliations:** 1 Semenov Institute of Chemical Physics, Russian Academy of Sciences, Moscow, Russia; 2 Institute of Mathematical Problems of Biology, Russian Academy of Sciences, Pushchino, Moscow Region, Russia; 3 Pushchino State University, Pushchino, Moscow Region, Russia; 4 National Research Center for Hematology, Russian Academy of Medical Sciences, Moscow, Russia; 5 Center for Theoretical Problems of Physico-Chemical Pharmacology, Russian Academy of Sciences, Moscow, Russia; 6 Department of Physics, Moscow State University, Moscow, Russia; 7 Institute of Mathematics, UMR 5208 CNRS, Universite Lyon 1, Villeurbanne, France; University of Glasgow, United Kingdom

## Abstract

**Background:**

Existence of flows and convection is an essential and integral feature of many excitable media with wave propagation modes, such as blood coagulation or bioreactors.

**Methods/Results:**

Here, propagation of two-dimensional waves is studied in parabolic channel flow of excitable medium of the FitzHugh-Nagumo type. Even if the stream velocity is hundreds of times higher that the wave velocity in motionless medium (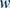), steady propagation of an excitation wave is eventually established. At high stream velocities, the wave does not span the channel from wall to wall, forming isolated excited regions, which we called “restrictons”. They are especially easy to observe when the model parameters are close to critical ones, at which waves disappear in still medium. In the subcritical region of parameters, a sufficiently fast stream can result in the survival of excitation moving, as a rule, in the form of “restrictons”. For downstream excitation waves, the axial portion of the channel is the most important one in determining their behavior. For upstream waves, the most important region of the channel is the near-wall boundary layers. The roles of transversal diffusion, and of approximate similarity with respect to stream velocity are discussed.

**Conclusions:**

These findings clarify mechanisms of wave propagation and survival in flow.

## Introduction

The number of biological systems with complex modes of excitation propagation is very large: blood coagulation [1], [2], excitable muscular systems [3], ecological systems [4], [5], neural tissue [6], etc. Many chemical and physical systems as well show complex spatio-temporal behaviour [7]–[10]. Propagation of excitation in many such systems can have the form of travelling pulses or trigger waves, which is typical for active media [11]–[13]. Despite the great variability, excitation spreading in these systems has many common properties. Therefore, use of the simplest models of active media played and still plays a vital role in the understanding of the mechanisms of excitation propagation in strongly non-equilibrium media. The model of FitzHugh-Nagumo is one of the simplest and the most widely used models of such systems.

Existence of flows and convection is an essential and integral feature for some of these systems, such as blood coagulation or bioreactors. For example, the stage of spatial propagation in blood coagulation occurs in a self-sustained manner [2], [14] because of the positive feedback activation of factor XI (the uppermost factor in the clotting cascade) by thrombin (the lowermost enzyme of the cascade). Both experiments and computer simulation show that flow can play a critical part in both the regulation of excitation threshold [15], [16] and the process propagation [17]. As the process occurs in flow, errors at this stage can result in pathological thrombus formation in the vasculature. There is an increasing number of problems, where flows define such processes as cell differentiation [18] or pattern formation in reaction-diffusion system in laminar flow [19], patterning of leaf veins [20], patterns arising from a combination of flow and diffusion in a two-dimensional (2D) reaction-diffusion system [21], in convectively unstable, oscillatory media [22] and many others.

Great variety of studied systems and differences in the experimental conditions lead in significant discrepancies between the results. In studies [19], [23], [24], a two-dimensional flow reactor was modeled, in which a self-sustaining reaction ran. The flow profile was assumed to be parabolic, with the velocity being largest at the channel axis and dropping to zero next to the channel walls. The reaction was initiated upon entry into the channel. Being influenced by flow, the flat reaction front became curved, this front advanced at a constant velocity, retaining its shape. Mathematically, the system was described with one partial differential equation. Numerical analysis of the model showed that stationary propagation of trigger waves is possible in a broad flow velocity range. The faster the flow, the more the reaction front is curved.

No stationary propagation of a plane wave, unless with wavefronts strictly along the stream lines, was observed in a two-dimensional active medium described with FitzHugh-Nagumo (FHN) equations (equations (2) below) [26]. The medium was assumed to be infinite and moving along the *x* axis at velocity 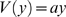, where *a* is constant. For a>a_*_, the excitation waves with initial orientation of the wave front orthogonal to the stream lines faded out.

It was shown in [25] that boundary conditions typical for blood clotting could arrest propagation of clotting in narrow vessels.

This discrepancy may be due to differences in the active media, flow types, and boundary and initial conditions in those studies. To understand the particular role of convectional and diffusional transfer, it was of interest to consider a simple model of active medium. In this study, we used a FHN model to analyze two-dimensional excitation waves running along the direction of a parabolic flow with the velocity 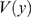 (see equation (1) below). The results of our numerical analysis are as follows.

1. Even if the stream velocity is hundreds of times higher that the wave velocity in still medium (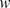), steady propagation of an excitation wave is eventually established, and its shape and velocity 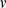 do not vary with time thereafter.
2. At high stream velocities, the steadily propagating excitation wave does not fill the channel completely, forming spatially localised excited regions, restrictons. They are especially easy to observe when the parameter values are close to the critical ones.
3. In the parameter region where no excitation wave exists in the still medium, a sufficiently fast stream is helpful for survival of steadily moving excitation (usually in the form of restrictons).

## Methods

### Mathematical model description

Let us consider a rectangular box of width *H* (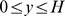) in the (*x*,*y*) plane, assuming that the medium is moving along the *x* axis at velocity *V*(*y*) with a parabolic velocity profile (corresponding to laminar flow of a viscous incompressible fluid):
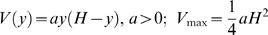
(1)


Let variables 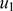 and 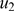 denote “activator” and “inhibitor,” respectively, in the FHN model. The equations describing wave processes in the channel then read:
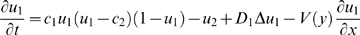
(2.1)

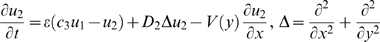
(2.2)


This set of equations differs from the classical FHN model in that both diffusion coefficients are assumed to be nonzero. Channel walls (horizontal boundaries) are assumed to be impermeable.

Parameters 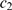, 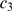, ε, *D*_1_, and *D*_2_ were fixed at the values used in [26]:
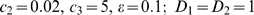
(3)


Parameters *a*, *c*_1_, and channel width *H* were varied in different numerical experiments. At parameter values (3), the excitable medium is monostable for 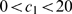 and *V*(*y*)≡0: it has a single stable spatially uniform state (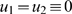), and a low excitation threshold. In the respective one-dimensional system, for 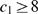, there are excitation pulses running at a constant velocity *w* without changing in shape: 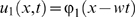, 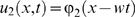. If the medium is not moving, the same formulas describe a solution to equations (2) in the form of a plane wave traveling along the channel. If we define the wave width 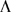 as the distance between level lines 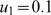 (for reference, 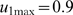), we obtain for 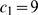 and the chosen parameter values that 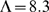 and the wave velocity 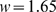. The values 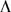 and 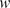 set the natural scales for length and velocity in this system.

Applying a perturbation to one channel end, we observe how an excitation wave subject to a stream is evolving. In numerical experiments, channel length *L* (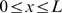) is chosen so large that its further increase does not change the results.

To initiate a wave at 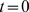, we set 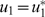 inside a narrow rectangle [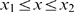, 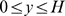] and 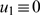 outside this rectangle (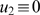 everywhere). If a perturbation is applied to the left boundary of the channel (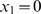), a wave arises that runs down the stream. If 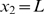, the wave runs up the stream. In numerical experiments, we employed a coordinate system moving in the positive *x* direction at velocity 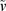. In other words, we transformed 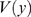 to 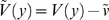, with 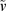 being chosen so as to have the stationary wave staying still (that is, it was taken equal to the wave velocity 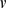 in the resting coordinate frame).

The following non-permeability boundary conditions were used on the channel walls:
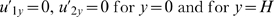
(4)


*Model solution.* For the numerical analysis of the model, the partial differential equations (2) were replaced with the difference equations. As in [23], we used alternating direction implicit method for differential items, and calculated explicitly the non-differential ones. Therefore, the difference scheme has the second order of approximation with regard to spatial variables *x* and *y*, and the first order with regard to time *t*. In order to find functions *u*(*x*,*y*,*t*) with acceptable accuracy, small intervals *h* should be used for *x* and *y*, and very small interval 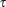 for *t*.

For steady-state processes, upon which this study is focused, the difference equations requirements can be significantly relaxed, because we use moving co-ordinates (substituting 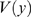 for 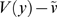). For most calculations, we used *h* = 0.1 and 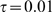. For comparison, characteristic length in the system is 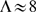, and charateristinc time is 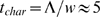 (see above). Therefore, the typical number of nodes in the calculations was of the order of 10^5^ (for *H* = 20 and *L* = 400). The typical time to achieve steady state for a travelling wave was 20–30 (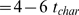), and the standard calculation time was from 0 to 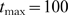. We had to increase the value of 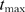 at near-critical parameter values; and 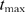 was also increased several-fold when we doubted that the found mode is a steady state. When necessary, the validity of conclusions was confirmed by control calculations with smaller *h* and 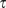.

## Results

The excitation waves in our study are autowaves: their shape and velocity in the steady-state mode do not depend on the excitation type. For example, it is possible to double the width of the initial excitation region (the difference 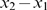, see the Methods section). This would not affect a steadily moving wave. A steady-state wave can fill up the channel completely (the excitation would then be present at all lines 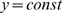) or only partly. In order to clearly distinguish between them, we shall henceforth use the term “wave” for all types of excitation propagation, while the term “restricton” will be reserved for isolated waves, which fill up the channel only partly as described below.

### Waves in flow

**Figure 1:**
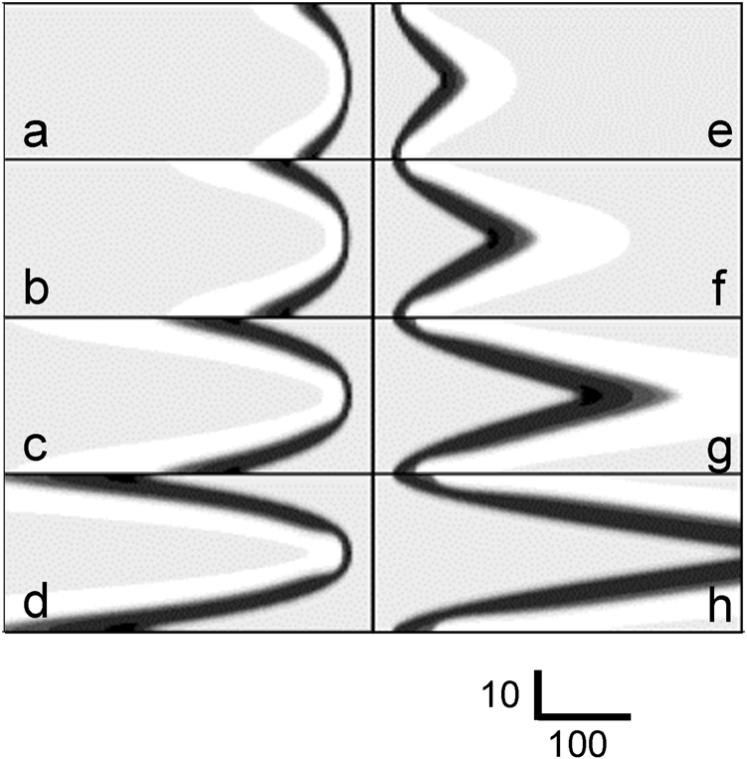
Effect of stream velocity on the shape of (a–d) downstream and (e–h) upstream waves, as calculated for 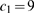, 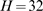, and 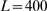: (a, e) 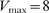, 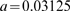; (b, f) 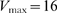, 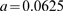; (c, g) 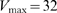, 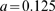; and (d, h) 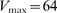; 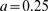. The stream direction is from left to right. Activator 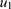 (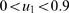) is shown on a nine-level gray scale, with white corresponding to 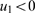. Note that the *x* and *y* axes are scaled differently (*y* axis is fivefold expanded relative to the *x* axis).

After a rather short transient period, steady-state excitation propagation is attained in the channel (Fig. 1). The front shape and velocity depend on the propagation direction. If the propagation direction coincides with the stream direction, the front edge of the wave resembles a parabola (Fig. 1a–d) whose vertex lies on the channel axis.

Parameters of a wave moving along the current depend on the flow velocity. The waves at low velocities are similar to those without flow in all respects. In the co-ordinates moving with the velocity 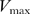, the wave velocity decreases with the increase of flow velocity as a square root of the maximal velocity with a proportionality coefficient of 0.1 at small flow velocities. At 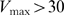, the dependence becomes more strong (Table 1). The influence of the flow velocity on the parameters of wave is most probably determined by tranversal diffusion (diffusion in the *y* direction). Increase of the forward front curvature coincides with the increase of the activator outflow across the current.

**Table 1:** Dependence of the wave velocity on the maximal flow velocity.

	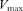	0	2	4	8	16	32	64	128
Wave along the current	(*v*-*V*_max_)*	1.6	1.5	1.4	1.3	1.2	1.0	0.8	0.2
Wave against the current	*v* **	−1.6	−1.0	−0.6	0.1	1.3	3.3	7.1	14.3

Parameters: 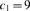, 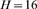, *V*_max_ = *aH* ^2^/4.

* The velocity of the wave moving along the current in relation to the maximal flow velocity.

** Negative velocity values mean movement against the current with regard to the channel walls.

The shape of the upstream wave is very different from that of the downstream one. The front edge consists of two curves meeting at a sharp angle on the channel axis. This wave is nearly motionless relative to the vessel walls (Table 1). In mid-stream, the wave velocity relative to the medium is approximately equal to the stream velocity but oppositely directed. At small flow velocities, the wave moves against the current even with regard to the channel walls. The wave is carried away along the current only when the flow velocity is ∼5-fold higher than the wave velocity in an immobile medium. In other words, in this case also, the wave velocity relative to the medium can be much higher than velocity 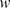: in Figs. 1d and 1h, 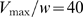. Although the Fig. 1a–d and Fig. 1e–h look very differently, they really are akin to each other in the sense that level lines of both variables in regions of fast excitation propagation relative to medium are tilted considerably, making a small angle with the *x* axis. Strikingly, steady-state propagation of the wave is achieved in a medium whose parts are moving at different velocities. We hypothesize that the shapes observed are such that diffusion coupling of adjacent areas of the wave allows the arising excitation structures to move as a whole.

### Two-dimensional wave as a combination of one-dimensional waves

Consider a stationary moving excitation wave for a fixed 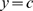. For each of these lines, we observe a one-dimensional excitation wave. If these waves were independent, their velocity with regard to the medium would be *w* (the velocity of a flat wave in the immobile medium), while velocities with regatd to channel walls would be *w*+*V*(*c*) or −*w*+*V*(*c*). Their width would be 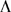 (the same for any c). However, one-dimensional waves along different horizontal straight lines are related to one another: in equations (2), 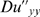 describes diffusion across the stream, which binds a set of independent one-dimensional waves into one excited area and determines its structure. With the increase of *V*_max_, the wave velocity with respect to the medium is decreased. This is particularly obvious in the channel axis (Table 1). Away from the channel axis, the wave front progressively curves (fig 1 left), giving rise to transversal diffusion of activator 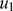 (in the direction from the channel axis to the wall). Transversal diffusion brings activator to adjacent lines earlier than the wave front carried by the stream comes there. Even small amounts of activator are sufficient to excite the medium. The more the front is oblique, the larger is the contribution from activator transversal diffusion, and the higher is the velocity of excitation propagation relative to the flowing medium. Thus, for downstream excitation waves, the axial portion of the channel is leading. In particular, it is this portion that determines the wave velocity. In other words, it can be said that the velocity of the wave is mostly determined by the processes around its most advanced part, convex in the direction of propagation.

**Figure 2:**
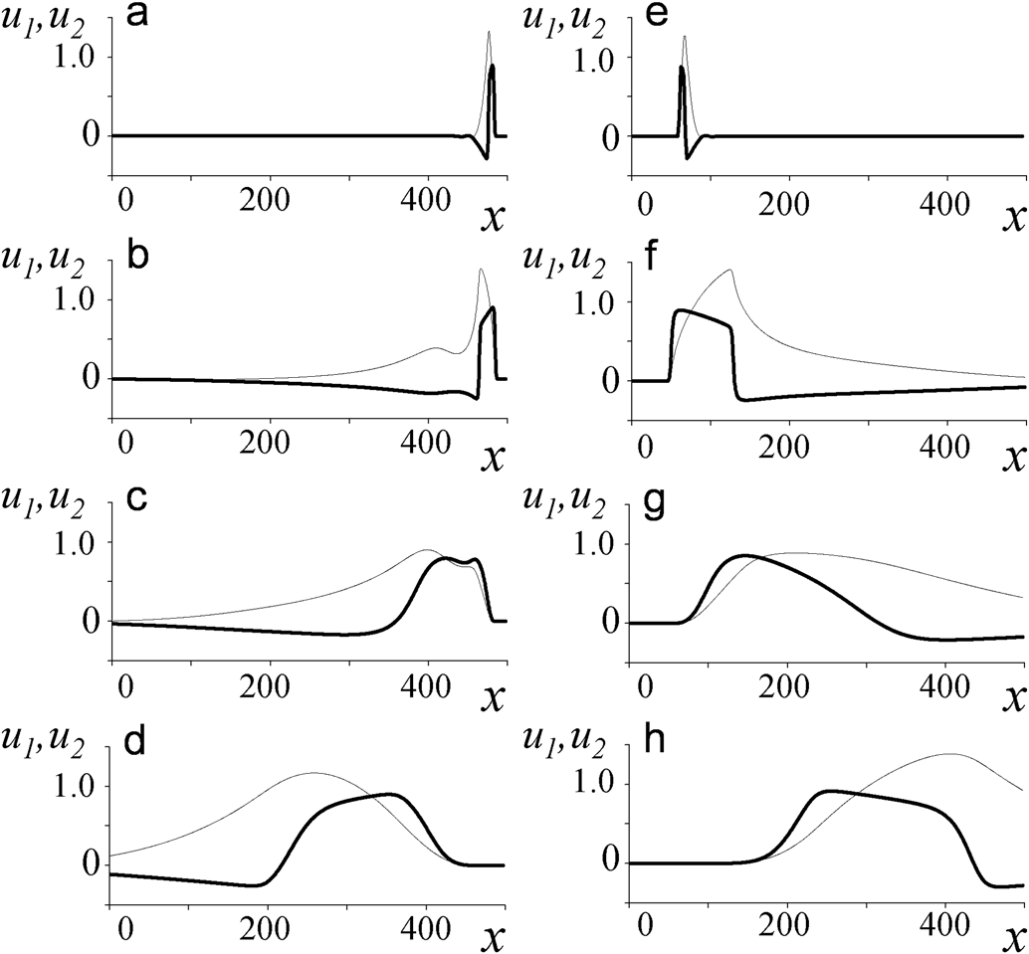
Longitudinal profiles of 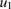 (thick lines) and 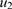 (thin lines) for downstream (left) and upstream (right) waves built for various distances across the channel. The stream direction is from left to right. In left panels, the distance across the channel is measured from the channel axis: (b) 0, (c) 3.4, and (d) 6.8. In right panels, the distance is measured from the channel wall: (f) 0, (g) 3.4, and (h) 6.8. For comparison, profiles of a flat waves moving from left to right (a) and from right to left (e) are shown. In calculations, 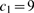, 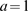, 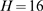, 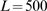; 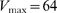.

The overall effect is due not only to one-dimensional waves near the channel axis. Those farther away from the axis also play a role. Reaching any given 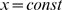 later, they support the excitation on the lines that are nearer to the channel axis. Therefore, the length of the excitation section on any fixed 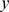 (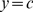) is greater than 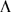, as can be clearly seen by comparing the profiles of the variables in still medium (Fig. 2a) with their profiles in flowing medium built at different distances from the channel axis (Figs. 2b–2d). This increase is likely due not only to the change of the wavefront inclination with regard to the axis of flow, but also to the increase of the length of the excited region along the direction perpendicular to the wavefront.

**Figure 3:**
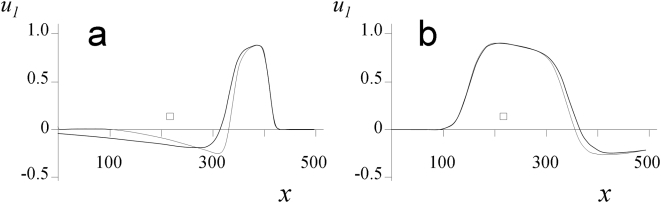
Comparison of plots 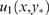 calculated for the parabolic (thick line) and modified (thin line) flow profiles: (a) downstream and (b) upstream waves. Here, 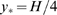 is the boundary between the preserved and changed parts of the flow. In calculations, 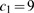, 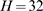, *L* = 500, and 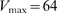. The stream direction is from left to right.

To ascertain the statement that the axial portion of the channel determines the wave velocity relative to the channel walls, we used two approaches: 1) compared the steady-state characteristics of excitation in the channel with a parabolic flow profile and in the channel with a composite profile following the same parabola from the channel axis to one quarter of the channel width and remaining constant thereafter (Fig. 3), and 2) increased the channel width.

Let the flow profile 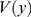 remain parabolic over the axial half of the channel (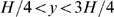), and become constant and equal to 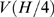 beyond the axial half. For the two profiles, we calculated values of 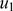 (“activator”) using the same calculation procedure and the same values of model parameters. In calculations, 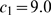. As an example, we present the results of one numerical experiment, in which channel width 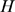 is 32, 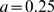, and 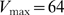. Comparing the results for the two flow profiles, we see that the steady-state wave velocities differ by less than 1%. Recall that the velocity of the downstream excitation wave is close to 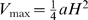.

As for the values of function 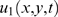 and 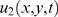, which are of interest to us, they are found in a numerical experiments for discontinuous range of independent variables: 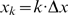 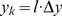, 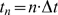. By fixing 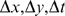, we compared values of 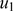 for the two flow profiles. The results for the same initial conditions for each set of discretization steps were the following.

1. Values on the channel axis (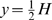), as well as on the line 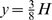, were nearly equal, the difference between the values calculated for the same 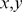 and 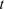 was less than 0.005.
2. The 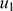 values on the boundary of the non-changed region, the line 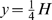, were quite similar at the front edge and differed significantly at the rear edge (Fig. 3a). The rear of the two-dimensional wave experienced the effect produced on the central region by the one-dimensional waves, which were “lagged” because of the stream.

Naturally, difference between values of 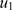 for the two calculations was great in the region where the flow profile has been modified (that is, for 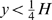 and 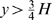).

In the second test, the parameter 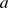 was fixed and the channel width was doubled. The 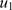 and 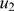 in the central half of the channel remained almost unchanged; there was only a four-fold increase in 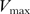 (velocity along the channel axis). The result is easy to explain because, if 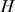 is changed into 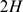 in equation (1), only a constant equal to 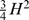 is added to function 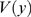 in the central half of the wider channel. The influence of the boundary conditions at the walls is small in the central region; the increase in the stream velocity by a constant only adds that same constant to the wave velocity.

### Upstream waves

For upstream waves (Fig. 1e–h), the central half of the channel is less important than for downstream waves, because activator diffuses away from there to already excited areas. In contrast, the important region is near the channel walls. Activator transversal diffusion from this region toward the channel axis provides for the concerted propagation of all one-dimensional waves. This behavior is observed even for very high 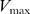; at high 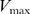, the steady-state wave velocity *v* (Table 1) is low, but the wave may move in the direction of the stream. Using the test like the first of the two described above, we have shown that the leading region in this case is the near-wall layer: a profound perturbation of the flow profile in the central half of the channel produced a negligible effect on the wave near the wall.

For upstream waves, the 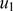 and 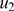 profiles along lines parallel to the channel axis are shown in Figs. 2f–2h. The nearer a line is to the wall, the more the profiles resemble their counterparts in the motionless medium. Away from the walls, the profiles do not change qualitatively; they only become more extended with increasing stream velocity (Figs. 2g, 2h).

Let the flow profile 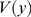 remain parabolic near the channel walls (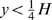 and 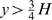), and become constant and equal to 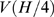 over the central half (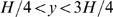). The numerical results for the two profiles in this case are analogous to those obtained with downstream waves. Again, in calculation, 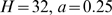
M.

1. The steady-state wave velocities calculated for the two profiles differ by less than 1%. It should be noted that the velocities of upstream waves are low.
2. The 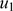 values on the channel wall (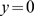), as well as on the line 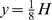, are nearly equal for the two calculations:, the difference between the values calculated for the same 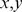 and 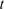 is less than 0.005.
3. The 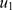 values on the line 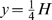 are quite similar at the front edge and different significantly at the rear edge (Fig. 3b), because the rear of the two-dimensional wave experiences the effect that “lagged” one-dimensional waves produce on the peripheral near-wall region.

Naturally, difference between values of 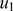 for the two calculations is great in the region where the flow profile has been modified (that is, for 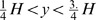).

Going over to discussing the second test, we write the velocity profile in the form:
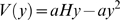



Doubling the channel width alters greatly the velocity profile near the wall 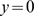. Varying 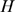, we can keep the stream velocity unchanged in the main (linear) term by maintaining 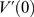, i.e., the value 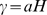. When the results calculated for 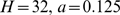 were compared with those calculated for 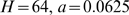, it appeared that the difference in the wave velocity was approximately 2%. In this experiment, doubling the channels width doubled the stream velocity along the channel axis and changed considerably the overall velocity profile (which remained parabolic). So, the velocity of the upstream wave is determined by the near-wall regions and depends mainly on the velocity gradient at the channel wall.

### Effect of the stream velocity

Figures 1a–1d show how the steady-state excitation shape varies with the stream velocity. The faster the stream, the more the excitation structure elongates in the stream direction. In numerical experiments, doubling the stream velocity nearly doubled the elongation in the *x*-axis direction. If the stream is very fast, the terms of the 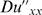 type in equations (2) are much smaller than the terms of the 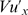 type, suggesting that the diffusion along the streamlines is insignificant. If we omit the terms containing second derivatives with respect to *x*, the problem acquires the following property of similarity. In the channel being considered, a *k*-fold increase in the stream velocity stretches out the profiles along the *x* axis by *k* times. However, longitudinal diffusion at the front edge of the wave is necessary for the propagation of the leading one-dimensional wave. Therefore, although approximate similarity with respect to parameter 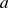 (or 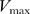) is observed over the most part of the excited area, there is no similarity in a narrow region near the front edge of the wave.

### Restrictons

The capacity of transversal diffusion to even out the wave velocities of adjacent areas is not infinite. After a stream velocity attains some critical value, a downstream wave can lose contact with the channel walls and turns into a “restricton”, that is, an excited structure moving at a constant velocity in the middle of the channel. This term was introduced in order to emphasize that excitation is spatially localized, restricted both along and across the channel axis. Control calculations employing densening grids over extended time intervals (the temporal interval exceeding that required for a system to achieve steady state by a factor of 10 and more) confirmed that restrictons are stable structures.

**Figure 4:**
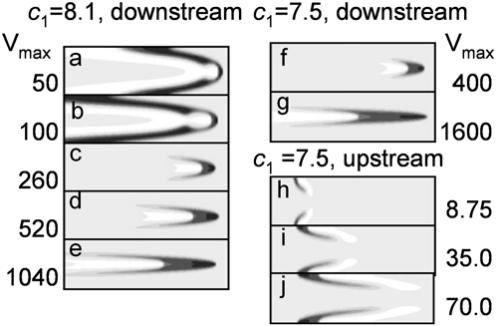
Effect of stream velocity on the evolution of (a–g) downstream waves and (h–j) upstream waves into restrictons, as calculated for (a–e) 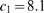 (above-critical value for which plane waves exist in the absence of flow), and for (f–j) 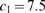 (subcritical value for which no plane waves exist in still medium). Note that, for subcritical 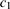, restrictons arise near the channel wall at low stream velocities (panels h–j). Activator 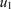 (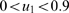) is shown on the same gray scale as in Fig. 1. In calculations, 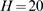 and 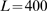. The *x* and *y* axes are scaled differently: *y* axis is eightfold expanded relative to the *x* axis.

The critical velocity depends on the “chemical” parameters of the system. With our choice of 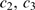 and 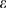 (see (3)), one-dimensional pulses exist for 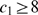. Near this critical value, the one-dimensional waves are “weaker” and more susceptible to external disturbances. In Figs. 4a–4c, one can see how the wave shape varies with increasing stream velocity for 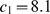. Waves are more complex in shape (cf. Fig. 1d with Figs.4a, 4b. In the leading region in the mid-channel, something like a nucleus develops: a zone of large 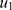 values (activator) surrounded on all sides with large 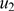 values (inhibitor). For 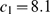, restrictons emerged in a rather wide channel at 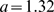 and existed throughout the stream velocity range used (Figs. 4c–4e). We also observed restrictons for *H* = 20, *L* = 800, and the stream velocity 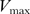 as large as 2000.

For 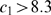, no restricton was generated even at stream velocities hundreds of times higher than the plane wave velocity *w* in still medium. Restricton solutions were also found for the waves moving against the current, but only within the region of parameters 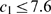 when waves do not exist in an immobile medium (Figs. 4h–4j). An upstream wave breaks down in the middle, giving rise to two slow restrictons that move as if being pressed against the wall. Their velocity with regard to the channel walls is small.

One of the major results of study [26] was that a flat front initially perpendicular to flow breaks when the velocity gradient exceeds the critical one. Appearance of restrictons in our calculations is also related to the breaking of that portion of the front, which is located in the maximal-gradient region. However, we were unable to find a direct correspondence between these phenomena. In our simulations, the value of the critical gradient strongly depended on the model parameters. At *c*_1_>8.3, restrictons did not appear even when the gradient (which is maximal near the channel walls) exceeded the critical value of [26] ten-fold and more.

### Bifurcation diagram

The emergence of restrictons and other phenomena described above essentially depend on the parameters of the stream and on the “chemical” parameters of system (2). We consider the effect of the latter taking parameter 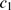 as an example.

**Figure 5:**
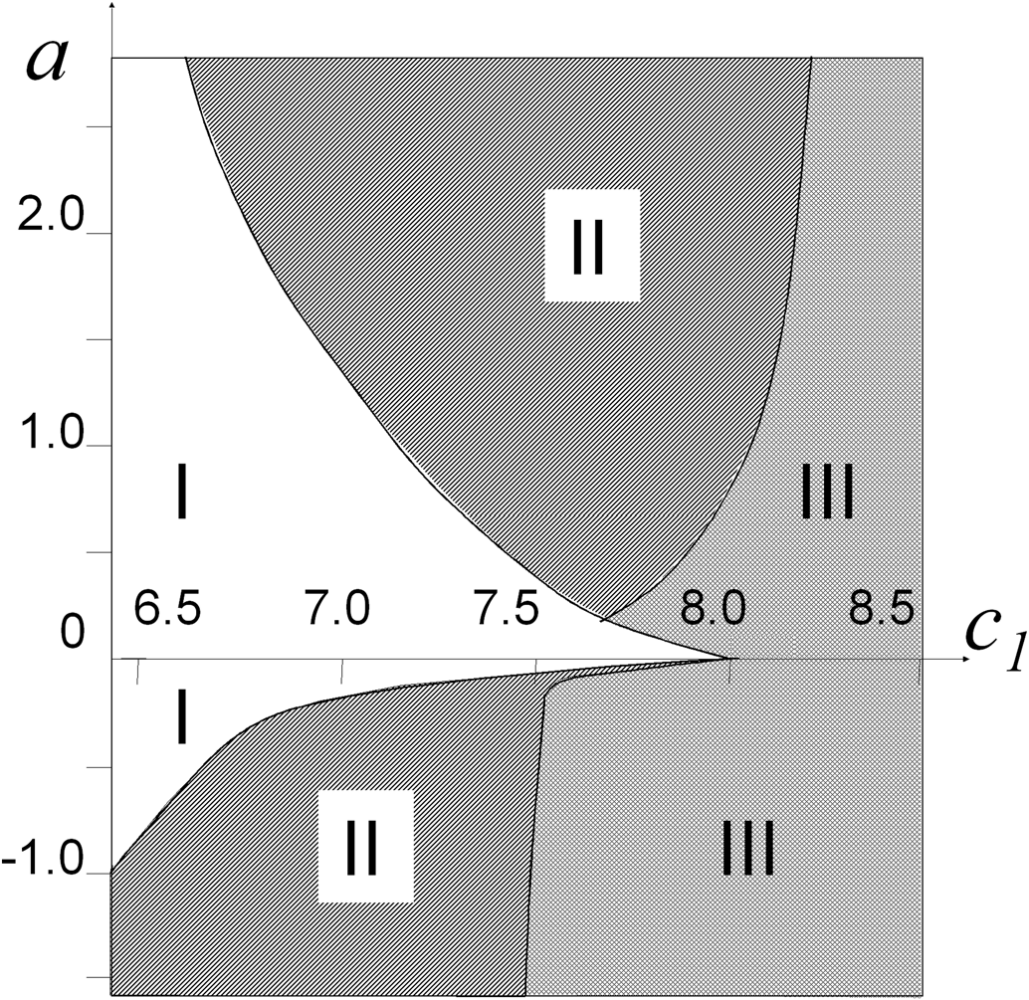
Bifurcation diagram of the model in the (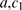) plane: (I) no wave solution exists because excitation vanishes rapidly; (II) restrictons; (III) waves. 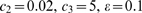
. Negative ordinate values correspond to upstream wave movement velocities. The calculations were performed for *H* = 16; it should be noted that the upper part of the diagram does not depend on *H*.

The diagram in Fig. 5 is composite; its upper section is for downstream waves, while the lower one is for upstream waves. In both cases, the calculations were performed as follows. The 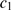 value was fixed, and the *a* parameter determining the flow velocity was changed gradually. For each *a*, we waited until stable state was achieved and monitored change of stable modes with the change of *a*.

### Downstream waves

**Figure 6:**
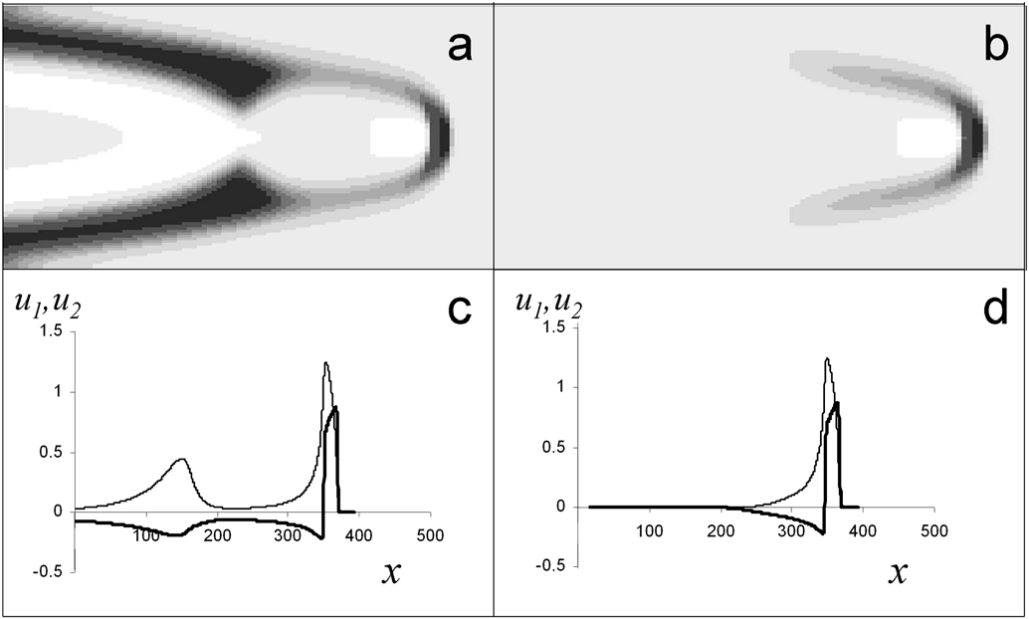
Restricton birth with increasing the stream velocity, as calculated for a 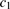 value close to a critical one (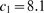): (a) 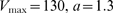; and (b) 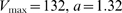. In calculations, 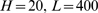. Profiles of 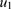 (thick lines) and 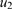 (thin lines) on the channel axis for (c) a downstream wave and (d) restricton that emerges at close values of the parameters, as calculated for (a) and (b), correspondingly. The stream direction is from left to right.

Figure 5 shows the {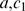} plane sectioning of the parameter space of solutions to model (2) (see eq. 1). In region I, the initial excitation rapidly vanishes. Region II corresponds to the existence of restrictons. For downstream waves increasing the stream velocity and crossing the boundary between regions II and III at fixed 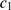, one would observe how the wave structure loses contact with the walls and gives rise to an restricton. One-dimensional pulses exist for 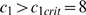. Near the critical value of this parameter, system (2) exhibits the richest behavior. For example, the 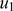 and 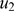 profiles along lines parallel to the channel axis may pass through two maxima, which is explained by the back effect of peripheral regions on more central ones: they feed the one-dimensional wave that have already begun to fade. The central part of the wave forms a nucleus from which wings extend to the vessel wall (Fig. 6a).

As the stream velocity increases, the wave loses contact with the walls, and only the central nucleus survives. The restricton that emerges (Fig. 6b) is in fact this nucleus. Their resemblance is clearly seen from a comparison of the *u_i_* profiles for a wave still touching the walls (Fig. 6c) and the restricton arising at a somewhat higher stream velocity (Fig. 6d).

The closer the “chemical parameter” 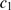 to its critical value, the lower the stream velocity is at which restrictons arise. As 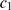 increases, the stream velocity at which restrictons emerge rapidly rises. For 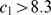, no restricton exists at any studied stream velocity. We should remind that flow appears to be a factor stabilizing waves. Restrictons were found in the parameter region where no excitation exists in still medium. Waves at 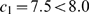 resembled restrictons arising at 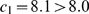 (cf. Figs. 4c, 4e with Figs. 4f–4g). There exists a small 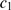 range (7.725–7.9), in which three types of behavior are observed with an increase in the stream velocity. At small 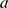, the excitation vanishes. At larger 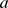, waves develop. A further increase in 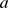 gives rise to restrictons.

An increase in the channel width produces little effect, if any, on the restricton shape if 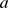 is kept constant. Actually, if 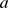 is the same, the flow profile in the central part of the channel does not change (only a constant is added to 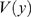).

Analysis of how the parameter diagram for downstream waves depends on the channel width has confirmed that the leading zone in this case is the excitation zone close to the channel axis (data not shown). There exist a channel width such that its further increase does not affect the diagram.

### Upstream waves

**Figure 7:**
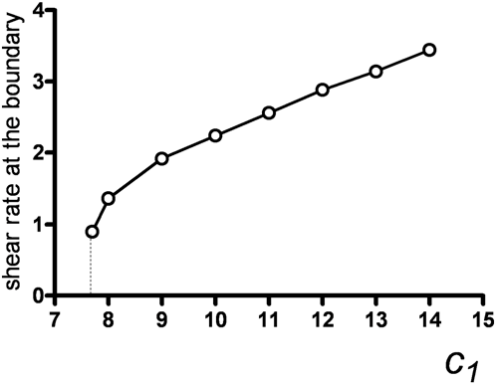
Dependence of wall shear rate, at which upstream wave is immobile with respect to the channel walls, on the 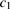 parameter. H = 16, 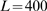. At 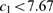, the excitation wave is always carried away by flow, and there is no such flow velocity, when the wave velovity is zero. Interestingly, at 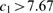, the dependence begins not at zero, but at a large gradient of flow velocity.

Upstream waves demonstrate some similarity with the dowstream waves upon changes in the “chemical” parameters, but this similarity is not strong (Fig. 5). The flow stabilizes upstream waves, as well as downstream ones. Flow results in the formation of stable steadily moving excitation waves at the same values of the parameter 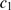., at which excitation in the immobile medium rapidly disappears. The border between the region where excitation disappears and the excitable region (Fig. 5, bottom part, border between region I and regions II, III), is achieved at higher flow velocities with the decrease of 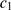. As for the downstream waves, restrictons appear with the increase of flow velocity at subcritical values of 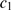. These small excitation regions near the borders do not resist the flow well and cannot move upstream, although their velocity is much smaller than maximal flow velocity. With the increase of flow velocity, the restrictons are stronger carried away by flow. At 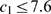, excitation cannot exist in the form of two restrictons and rapidly disappears in the middle of the channel. For 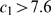, restrictons do not appear at all, and stable excitation wave appears (III). The ability of the wave to move against the current increases with the increase of 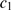. This demonstrates the dependence of the wall shear rate when the upstream wave is immobile with regard to the wall (Fig. 7). At 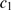 values close to 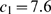, this dependence is strongly non-linear (Fig. 7); however, when 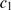 exceeds 8, it becomes almost proportional to 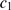.

## Discussion

The relationship between the wave velocity and the stream velocity in our study is similar to the relationships described in the cited studies [19], [23], [24], which consider trigger waves in the model of one variable. Setting *c*_3_ to zero in equations (2), we reduce them to one equation. If 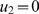 at 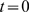, no inhibitor would be generated in the system: 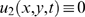. With *c*_2_ also set to zero, we come to the equation similar to that considered in [19], [23], [24]. However, with a nonzero excitation threshold (unlike zero in the cited studies) and diffusion of both variables, wave phenomena in our study are more diverse and complex.

The shape of the front edge depends mostly on activator transversal diffusion (Fig. 2). Inhibitor increases more slowly and produces little effect on the front edge of the wave. This influence of transversal diffusion is likely to be a general phenomenon for all excitable media ***in the presence of flows***. The same influence likely defines that the presence of flow increases stability of excited structures: both waves and restrictons are observed at such values of system parameters, when the system without flow cannot be excited. While it seems reasonable to assume that the stabilizing effect of flow on excitation is due to shape change and transversal diffusion, the mechanism of this interesting phenomenon requires further elucidation.

The most interesting and immediate application of the obtained results can be in the understanding of the regulation mechanisms of blood coagulation. In our opinion, of particular interest in this respect are the following results: formation of restrictons, and ability of flow to allow wave propagation even when waves do not exist in immobile medium. In the vascular system, there is a wide range of wall shear rates from zero up to 2000 s^−1^; this means that conditions appropriate for almost all modes of wave propagation can be found. Normal clotting is usually effectively localized at the site of damage by specific mechanisms [14], but this can be not so in pathology. And, in such cases, it is of interest that the self-sustained mechanisms of clotting may lead to failures of mechanisms limiting thrombus propagation, which could assume the form of restrictons, or flow-assisted autowave survival. However, specific predictions about these processes and can be done only with detailed mechanism-driven models of blood coagulation.

It should be stressed, however, that blood coagulation is an extremely complicated process, and blood itself is a non-Newtonian fluid. While there are indications that the findings of this study obtained using a simple model of an active medium and parabolic flow are of general nature and are retained for other systems and flow profiles, specific predictions for concrete systems such as coagulation should be done using much more detailed and mechanism-driven models accounting for the complexity of biochemical reactions and hydrodynamics [27], [28], [29]

The study of Ermakova et al. [25] has shown that one of the most important factors limiting the propagation of coagulation wave is ability of vessel walls to inhibit the process. However, possibility of restricton solutions, when excited region occupies the central part of the vessel and is not in contact with vessel wall, is a source of danger that this excitation will not remain localized. Therefore, it is of great interest to learn if restricton modes of clot formation are possible in blood and which parameter changes lead to these solutions.

Current knowledge of coagulation is detailed, and adequate mathematical models of the process have been developed. This makes theoretical analysis of possibility and region of existence of restricton solutions possible.

We experimented with a parabolic flow; however, it is clear that qualitatively similar results would be obtained for other profiles that, like a parabolic one, have one maximum and decline to zero at the channel walls. Shear flow profiles of this kind are quite widespread.

We described waves that evolved in flowing medium from a localized perturbation over the entire channel cross section (see model description). It may well be that there are also other steady-state solutions to model (2). This question has yet to be addressed in future studies.
